# Characteristics of large vestibular aqueduct syndrome in wideband acoustic immittance

**DOI:** 10.3389/fnins.2023.1185033

**Published:** 2023-05-25

**Authors:** Ao Li, Haoliang Du, Junyan Gao, Yuqin Xu, Ning Zhao, Song Gao, Yuxuan Xing, Xiaoyun Qian, Xia Gao, Ye Yang

**Affiliations:** ^1^Jiangsu Provincial Key Medical Discipline (Laboratory), Department of Otolaryngology-Head and Neck Surgery, Nanjing Drum Tower Hospital, The Affiliated Hospital of Nanjing University Medical School, Nanjing, China; ^2^Research Institute of Otolaryngology, Nanjing, China; ^3^Department of Technical Counseling, Jiangsu Children’s Rehabilitation Research Center, Nanjing, China

**Keywords:** large vestibular aqueduct syndrome (LVAS), wideband acoustic immittance, maximum absorbance, mean tympanogram, frequency-absorbance curve

## Abstract

**Objective:**

To describe the characteristics of large vestibular aqueduct syndrome (LVAS) in wideband acoustic immittance (WAI) and to explore whether inner ear deformity has an impact on WAI results.

**Methods:**

Subjects with typical LVAS (LVAS group) and control subjects with a normal anatomical structure of the inner ear (control group) were screened from pediatric patients with cochlear implants using thin-slice computed tomography (CT) images of the temporal bone. With inflammation of the auditory canal and middle ear excluded by routine ear examination and 226 Hz acoustic immittance, WAI data were acquired. Then, the maximum absorbance as the major observation indicator on the mean tympanogram was compared between the LVAS group and control group, and a descriptive comparison of the mean tympanogram and frequency-absorbance curve at peak pressure was performed between the two groups.

**Results:**

The LVAS group included 21 cases (38 ears), and the control group included 27 cases (45 ears). All LVAS subjects met the Valvassori criteria, and the VA at the horizontal semicircular canal displayed flared expansion. On the mean tympanogram, the maximum absorbance in the LVAS group (0.542 ± 0.087) was significantly higher than that in the control group (0.455 ± 0.087) (*p* < 0.001). The tympanogram in the LVAS group showed an overall elevation, and the absorbance at all pressure sampling points was significantly higher than that in the control group (*p* < 0.001). The frequency-absorbance curve at peak pressure first increased and then decreased in both groups, and the LVAS group showed higher absorbance than the control group in the frequency range below 2,828 Hz. The absorbance at 343–1,124 Hz was significantly different between the two groups (*p* < 0.001), and 343–1,124 Hz was the major frequency range at which the maximum absorbance on the mean tympanogram increased in the LVAS group.

**Conclusion:**

Large vestibular aqueduct syndrome (LVAS) shows increased absorbance in low and medium frequency ranges in WAI. The maximum absorbance on the mean tympanogram can serve as a reliable evaluation indicator. Inner ear factors must be considered when middle ear lesions are analyzed by WAI.

## Introduction

Wideband acoustic immittance (WAI) is a novel middle ear clinical assessment tool to reflect the functional status of the middle ear sound transmission system by measuring the sound energy back to the external auditory canal through the tympanic membrane. WAI has been found to be more sensitive to lesions of the middle ear sound transmission system and can effectively avoid indistinct characteristics of diseases or even missed diagnoses resulting from the offsetting of acoustic compliance in complex lesions in the middle ear, reflecting significant advantages over traditional 226 Hz acoustic immittance (Sanford et al., 2013; Liu et al., 2021; Miehe et al., 2022). WAI are better indicators of conductive hearing loss and middle-ear dysfunction relative to standard 226 Hz tympanometry. The use of a single frequency as in conventional tympanometry cannot investigate middle-ear function at all the frequencies which are critical for human auditory communication. WAI can provide references for the analysis and identification of middle ear diseases and has gradually attracted clinical attention in recent years. During sound transmission, however, sound energy conduction disorders can also be caused by hydrodynamic changes in the inner ear lymphatic fluid, which manifest as conductive hearing loss (Madden et al., 2003). Deficits in the cochlea can also affect middle ear functions. While approaching to patients with inner ear malformations, it is significant to consider their hearing characteristics. Therefore, whether the inner ear components must be considered when analyzing WAI results is worth exploring.

The large vestibular aqueduct syndrome (LVAS) is characterized by the presence of an abnormally large vestibular aqueduct (LVA) generally associated with fluctuating, progressive sensorineural hearing loss (SNHL), often with sudden, stepwise onset or progression secondary to activities involving minor head trauma, large sudden shifts of barometric pressure, and the Valsalva maneuver (Nakashima et al., 2000; Ding et al., 2021; Ota et al., 2021). Large vestibular aqueduct syndrome is one of the most frequent morphogenetic contributors to hearing loss in children, although its prevalence is underestimated. In addition, it is often associated with other congenital inner ear anomalies, the most common being an abnormally large vestibule, an enlarged semicircular canal, or a hypoplastic cochlea (Kei et al., 2013). With large vestibular aqueduct syndrome (LVAS), a typical kind of inner ear deformity, as an example, whether structural deformities generate characteristic manifestations in WAI and the impact of anatomical deformities of the inner ear on WAI absorbance were analyzed in this paper.

## Materials and methods

### Clinical data

**FIGURE 1:**
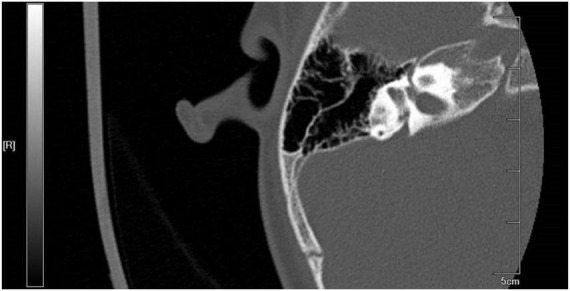
Computed tomography (CT) imaging from recruited large vestibular aqueduct syndrome (LVAS) Patient.

**FIGURE 2:**
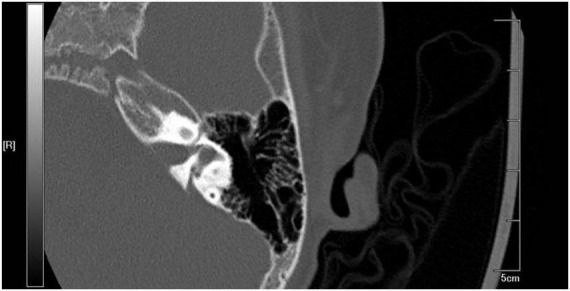
Computed tomography (CT) imaging from recruited large vestibular aqueduct syndrome (LVAS) Patient.

Pediatric patients who received cochlear implantation in Nanjing Drum Tower Hospital from July 2019 to June 2022 (LVAS group) were recruited. The inclusion criteria were as follows: (1) patients who met the computed tomography (CT) imaging diagnostic criteria (Valvassori criteria) for LVAS [i.e., a diameter from the crus commune of the semicircular canal to the first part of the outer orifice of the VA (midpoint measurement, MP) ≥ 1.5 mm and a diameter of the outer orifice of the VA (operculum measurement, OP) ≥ 2 mm on axial high-resolution CT (HRCT) images of the temporal bone] and (2) patients with unobstructed external auditory canals, intact tympanic membranes on otoscopy, normal middle ear development, and no inflammatory lesions on thin-layer CT images of the temporal bone. Actual CT images from the recruited patients were demonstrated in Figures 1, 2.

The exclusion criteria were as follows: (1) patients complicated with inner ear deformities, such as Mondini malformation or structural abnormalities of the internal auditory canal and (2) patients with a recent history of upper respiratory tract infection or a non-type A tympanogram. Finally, a total of 21 patients (38 ears) met the inclusion and exclusion criteria, including 13 males and 8 females with a median age (interquartile range) of 57 months (35, 96). In addition, pediatric patients of similar ages who received cochlear implant surgery during the same period were recruited as the control group. A total of 27 patients (45 ears) met the inclusion criteria (normal anatomical structure of the cochlea on thin-layer CT images of the temporal bone, no recent history of upper respiratory tract infection, and a type A tympanogram), including 14 males and 13 females with a median age (interquartile range) of 32 months (21.5, 58).

### Assessment methods

#### CT scan of the temporal bone

Computed tomography (CT) scanning of the temporal bone as a routine preoperative examination was conducted using a GE LightSpeed 64-slice spiral CT scanner with the following parameters: slice thickness of 0.45 mm, slice gap of 0.45 mm, pitch of 0.562, 140 kV, and 300 mAs. After image acquisition by bone algorithms, the image was reconstructed bilaterally with the promontorium tympani as the center, and the reconstruction interval was 0.3 mm. In the best plane displayed on the cross-sectional image, the external orifice diameter of the VA (ODVA) inside the ES bone and the midway diameter of the VA (MDVA) (i.e., the diameter of the aqueduct between the midpoint of the crus commune of the vestibulum and the external orifice of the VA) were measured.

#### WAI

Wideband acoustic immittance (WAI) was conducted on the patients in a quiet state using the Titan WAI meter (Interacoustics, Denmark) in a soundproof room with a background noise < 30 dB (A). The patients were sedated with chloral hydrate, and the external auditory canal and tympanic membrane were assessed prior to the test. Cerumen, if present, should be removed. The test software included 226 Hz acoustic immittance (initial pressure: +200 daPa, end pressure: −400 daPa, positive → negative) and WAI (mixed short sound with an intensity of 85 dB SPL and a frequency of 226–8,000 Hz). The test probe oriented toward the tympanic membrane was plugged into the external auditory canal, and after the sealing test was passed, the start button was clicked to automatically perform 226 Hz acoustic immittance, WAI, and the stapedius reflex test. Then, 3D images were generated by the software according to the absorbance at different pressure and frequency sampling points. Based on the resulting images, frequency-absorbance curves at peak pressure and ambient pressure (226–8,000 Hz, 1/24 octave sampling) and the 375–2,000 Hz mean tympanogram (sampled every 1 daPa at +200 daPa to −400 daPa) were generated from different analysis perspectives, and the sample data were generated in Excel.

The data for subjects who met the inclusion and exclusion criteria were identified, and the maximum absorbance on the mean tympanogram was used as the major indicator for analyzing the characteristic manifestations of LVAS in WAI. A descriptive analysis was conducted on the mean tympanogram and frequency-absorbance curves at peak pressure.

### Statistical methods

Normally distributed continuous data are described as the mean ± standard deviation and compared between two groups by the *t*-test. Non-normally distributed data are described as the median (quartile) and compared between two groups by a non-parametric test. Finally, qualitative data are described as numbers and percentages and compared between two groups by the chi-square test or Fisher’s exact probability test. *P* < 0.05 was considered statistically significant (SPSS 22.0, Chicago, IL, USA).

## Results

**FIGURE 3:**
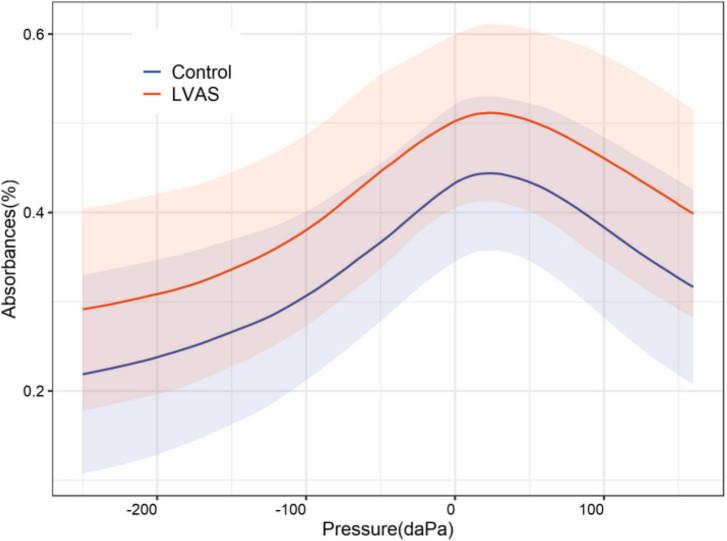
2-dimensional comparison of the mean tympanograms between the large vestibular aqueduct syndrome (LVAS) and control groups.

The mean tympanogram was unimodal in both groups. The maximum absorbance in the LVAS group (0.542 ± 0.087) was significantly higher than that in the control group (0.455 ± 0.087) (*p* < 0.001). The pressure point where the maximum absorbance was located showed no statistically significant difference between the two groups [LVAS group: (16.079 ± 43.202) daPa vs. Control group: (18.756 ± 29.949) daPa, *p* > 0.05]. The absorbance at each pressure sampling point in the LVAS group was higher than that in the control group, and the difference was statistically significant (*p* < 0.05 or 0.001) (Figure 3).

**FIGURE 4:**
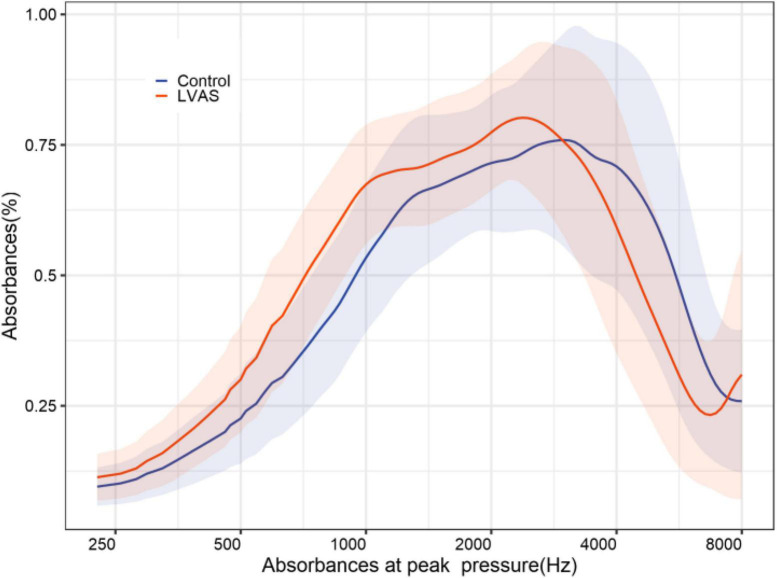
2-dimensional comparison of frequency-absorbance curves at peak pressure between the large vestibular aqueduct syndrome (LVAS) group and the control group.

**FIGURE 5:**
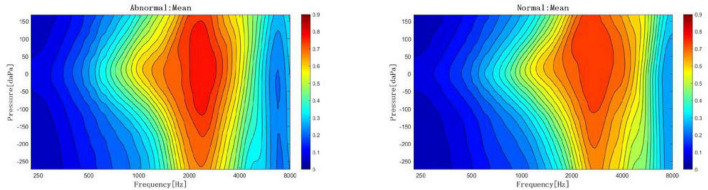
2-dimensional comparison of frequency-absorbance curves at peak pressure between the large vestibular aqueduct syndrome (LVAS) group and the control group (**left**: control group; **right**: LVAS group).

**FIGURE 6:**
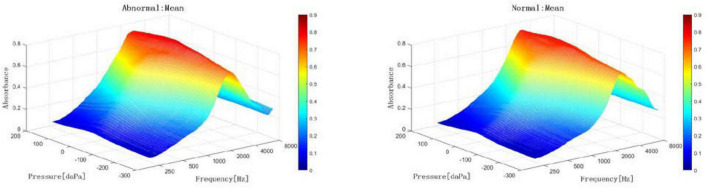
3-dimensional comparison of frequency-absorbance curves at peak pressure between the large vestibular aqueduct syndrome (LVAS) group and the control group (**Left**: control group; **right**: LVAS group).

With increasing acoustic frequencies, the frequency-absorbance curve at peak pressure first increased and then decreased in both groups. In the LVAS group, the curve sharply increased at 226–1,029 Hz and slowly increased at 1,029–3,084 Hz. In the control group, the curve sharply increased at 226–1,373 Hz and slowly increased at 1,373–3,174 Hz. The LVAS group had higher absorbance than the control group in the frequency range below 2,828 Hz, while the results were the opposite in the frequency range above 2,828 Hz. In particular, the absorbance at 343–1,124 Hz, 1,943–2,448 Hz, and 3,886–6,727 Hz showed statistically significant differences between the two groups (*p* < 0.05 or 0.001) (Figures 4–6).

## Discussion

As a common structural deformity of the inner ear, LVAS originates from developmental disorder of the endolymphatic ducts (endolymphatic sacs) at mid-embryo stages, which can maintain the VA and endolymphatic ducts (endolymphatic sacs) in a wide state at early-embryo stages until LVAS occurs after birth (Hunter et al., 2013; Shahnaz et al., 2013; Scheperle and Hajicek, 2020; Eberhard et al., 2022a). Therefore, to better analyze the impact of inner ear structural abnormalities on WAI absorbance, cases were strictly selected according to the Valvassori criteria, i.e., the subjects needed to meet the original two criteria, and the VA on the image should demonstrate flared expansion.

The mean tympanogram of WAI reflected the superposition of the tympanograms in the 3D absorbance diagram at an acoustic frequency of 375–2,000 Hz. Since absorbance was used as the measurement indicator, the unpredictability of the diagram of immittance caused by mutual changes in elastic stiffness and mass with increasing acoustic frequency could be avoided, which is an advantage of WAI. Moreover, the absorbance value corresponding to the pressure axis in the diagram was an average and contained more robust information than the measured value at a specific sampling point in the frequency-absorbance curve. Furthermore, the resulting diagram was similar to a traditional 226 Hz tympanogram (Sun, 2016; Aithal et al., 2019). With reference to the principle of Liden-Jerger classification based on the value and location of the maximum immittance, the maximum absorbance on the mean tympanogram was selected as the major evaluation indicator, which could be better quantified in a diagram analysis (Fu et al., 2017). Additionally, a descriptive analysis was conducted on the change trend and difference between the overall diagram and the diagram in the control group. Finally, the frequency ranges that contributed to the maximum absorbance in the frequency-absorbance curve were analyzed. As a result, the data could be analyzed comprehensively.

In this study, the maximum absorbance in the LVAS group was significantly higher than that in the control group, and the absorbance at all pressure sampling points on the mean tympanogram showed a significant increase. That is, the absorbance curve on the mean tympanogram showed an overall elevation compared to that in the control group. Furthermore, the pressure point where the maximum absorbance was located on the mean tympanogram corresponded to the frequency-absorbance curve at the peak pressure. The absorbance in the frequency range < 2,500 Hz was higher in the LVAS group than in the control group, which was consistent with the frequency superposition range of the maximum absorbance. The absorbance in the frequency range < 1,100 Hz was significantly different between the two groups, which was the major frequency range where the maximum absorbance increased (Aithal et al., 2017; Demir et al., 2020; Eberhard et al., 2022b; Meng et al., 2022; Tanno et al., 2022). At the same time, the absorbance in the frequency range > 3,000 Hz in the frequency-absorbance curve at peak pressure was lower in the LVAS group than in the control group. The influencing factors for middle ear sound transmission include elastic stiffness and mass. Elastic stiffness has a greater impact on the transmission efficiency of low-frequency sound signals, while mass has a greater impact on the transmission efficiency of high-frequency sound signals (Kaya et al., 2020; Downing et al., 2022; Miehe et al., 2022; Aithal et al., 2023). The available results are believed to be related to changes in the anatomical structure and biochemical properties of LVAS and the frequency of the stimulus sound. Although the pathogenesis of LVAS remains unclear, many theories have been proposed. Anatomically, the internal orifice of the VA is located on the medial wall of the vestibulum adjacent to the oval window, which is normally a tiny bony canal. Previous studies reported that structural expansion would lead to the expansion of the endolymphatic ducts and endolymphatic sacs. This increases the pressure of the endolymphatic and perilymphatic fluids, leading to a higher elastic stiffness. As a result, this physical change increases low-frequency sound conduction efficiency (Özdemir et al., 2022).

In addition, excessive pressure can cause rupture of the membrane labyrinth, ion mixing of endolymphatic and perilymphatic fluids, and stria vascularis dysfunction, resulting in ion transport disorders and protein exudation. Low signals on inner ear images indirectly indicate changes in the composition and properties of lymph fluids (Polat et al., 2015; Stuppert et al., 2019; Hougaard et al., 2020; Wu et al., 2022). These changes can damage hair cells and alter sound transmission performance. Due to the above factors, the absorbance of low-frequency sounds on the mean tympanogram may increase, particularly an overall increase in the entire pressure range. On the other hand, a high-frequency sound has a short wavelength, and its energy decays quickly during transmission, which is more susceptible to mass. Due to the VA enlargement, the volume of the inner ear increases. This reduces the conduction of high-frequency sound (Sundgaard et al., 2022), i.e., a decline in absorbance, suggesting that an increased inner ear volume should be part of the mass in the whole sound transmission system. From the audiology perspective, LVAS often manifests as characteristic low-frequency air-bone conduction differences, but no reports are available on air-bone conduction differences in patients with high-frequency residual hearing, probably suggesting a difference in the absorbance of low-frequency and high-frequency sounds. Of course, the present results remain to be further validated by more studies.

### Limitations of our study

The small sample size of our study may have reduced our ability to provide WAI norm data for patients with LVAS. Therefore, studies with a larger sampling size are strongly recommended. Additional work is needed to determine normative data for various age groups since age-related differences in ambient and tympanometric WAI have been observed. Moreover, key indicators should be identified to develop high sensitivity and specificity in WAI analysis.

## Conclusion

In conclusion, LVAS shows increased absorbance in WAI’s low and medium frequency ranges. The maximum absorbance on the mean tympanogram can serve as a reliable evaluation indicator. Inner ear factors must be considered when middle ear lesions are analyzed by WAI. In the future, whether this conclusion applies to other inner ear deformities will be further explored.

## Data availability statement

The raw data supporting the conclusions of this article will be made available by the authors, without undue reservation.

## Ethics statement

The studies involving human participants were reviewed and approved by the Nanjing Drum Tower Hospital. Written informed consent to participate in this study was provided by the participants’ legal guardian/next of kin.

## Author contributions

AL: conceptualization, methodology, formal analysis, visualization, and writing—original draft. HD: data curation, methodology, formal analysis, investigation, and writing—original draft. JG: formal analysis, investigation, resource, and data curation. YXu: formal analysis, investigation, and resource. NZ: data curation and investigation. SG: resources and investigation. YXi: software and validation. XQ and XG: supervision, project administration, writing–review, editing, and resources. YY: project administration, writing–review, editing, and resources. All authors contributed to the article and approved the submitted version.
